# Qualitative and mixed methods research in trials

**DOI:** 10.1186/s13063-015-1084-4

**Published:** 2015-12-08

**Authors:** Claire Snowdon

**Affiliations:** London School of Hygiene and Tropical Medicine, London, UK

This BMC Special Issue on qualitative and mixed methods research in clinical trials marks an important stage in both methodological development and collaborative relationships between researchers from different academic and clinical fields. It is indicative of the level of interest in the work of a now-substantial community of researchers, and a measure of the extent of integration of that work into the methodological component of trials. It highlights the ability to explore, explain and work through important questions for trials, as well as to provide trialists with evidence-based tools which they can use to improve and refine their own research practice. It is a sign of the growth in comfort and familiarity within the trials community with the aims, perspectives and techniques of qualitative and mixed methods research.

Key aims of this series are to showcasethe range of qualitative and mixed methods research in this field and the ways in which they can be utilised to support the methodological growth of clinical trials. The range of qualitative and mixed methods studies published in *Trials* in recent years is indicative of the growing importance of these approaches. Now endorsed by funders [3] and embraced by trialists [16], qualitative studies are moving beyond being an adjunct to a trial or an opportunist form of study. They are becoming an integral activity for trial teams and clinical trials units [19, 2]. This is a long way to have come in the relatively short history of this particular field. In the late 1980s and 1990s, early work on views of trials and their processes was often hypothetical and adopted simple thematic approaches, commonly collecting data with simple questionnaires. It often focused on a small number of key logistical and ethical issues: recruitment, standards of consent and challenges of integrating research and clinical obligations. From this has grown a complex field with expanding interests and objectives using a range of methodological approaches.

If we look at the field now, those core concerns are still important with research around recruitment still forming a large part of the qualitative and mixed methods portfolio. This strand of work continues because, as with the trials we study, it is rarely the case that there can be one definitive piece of research which fully answers our questions. Many studies seek to better understand the challenges involved in different settings (see for example work by Donovan and colleagues [6]) and are doing so with increasing sophistication. Some studies, for instance, involve direct intervention in an ongoing trial as the knowledge gained in pilot or integrated qualitative research is fed into the recruitment process to improve practice [7, 13]. Such studies provide a depth analysis of one trial, seeking to understand the particular circumstances in which it is set and the conditions under which it operates, in order to implement change, but these studies almost always have lessons for upcoming trials. New trials bring with them new challenges, and we push on with recruitment-related research as the questions as well as the settings keep on changing. We now have, for instance, the opportunity to recruit hard-to-reach research populations via social media [24]. We have legislation in Europe which permits trial enrolment in emergency settings without prior consent. Qualitative research helps us to understand how such approaches to recruitment in these novel settings are viewed [21, 25–27]. Studies of decision-making, consent and recruitment are part of an ongoing endeavour to chip away at important questions and improve understanding of the multifaceted and dynamic nature of this key area of trials practice. The body of qualitative research on recruitment is now sufficiently large for reviews [10] and meta-analyses to be a reality [9, 11], and given the importance of these approaches, we are working with our sister journal *Systematic Reviews* for this Special Issue (see *Systematic Reviews* Special Issue editorial by David Gough).

Ethical aspects of trial conduct also continue to be an important area for qualitative and mixed methods research. Exploration of the impact of policies such as feedback of results, a policy driven by development of fair and transparent trial processes, demonstrates diverse reactions among participants and, for some, profound and enduring effects made visible only through qualitative inquiry [5, 20]. The groundwork of such studies in identifying ethical issues, benefits and concerns allows us to move on to develop the tools and evidence base and for change. The Ottawa Statement on the Ethical Design and Conduct of Cluster Randomized Trials [28], based on a complex mixed methods study, is a recent example of how we can progress directly from qualitative research to the generation of evidence-based guidelines for the good conduct of clinical trials.

The Ottowa Statement is a positive example of the value of holistic consideration of the design and analytical choices trial teams make, issues which are not so often considered by qualitative researchers. This is an important new direction for study with scope for qualitative exploration of the value of different trial methods and methodological questions (see, for instance, the work of Dimairo and colleages [4] on trialists’ views of adaptive trial designs and Snowdon and colleagues [20] on expert methodologists’ views of inclusion and exclusion criteria in high mortality trials). Views and experiences of the effects of design on trial participants are also important but have received insufficient scrutiny. Work in this area is, however, emerging with, for instance, studies of views of the use of sham interventions [18], placebos [23] and control groups [12]. Qualitative and mixed methods researchers are now demonstrating the contribution they can make to the development of trial outcome measures which are conceptually grounded in the knowledge, values, preferences and needs of patients, trial participants and healthcare providers [8, 14].

We need to continue to grow in our understanding of the value and application of qualitative research in this context, especially in mixed methods studies where integration of data from different approaches is a crucial and complex endeavour [15]. We need to engage with the epistemological challenges involved [1]. The qualitative stream of work at ConDuCT (Collaboration and Innovation for Difficult or Complex Randomised Controlled Trials - http://www.bris.ac.uk/social-community-medicine/centres/conduct2/research/theme-2.html), one of the MRC Hubs for Trial Methodology Research, and the QUART study to consider the contribution of qualitative research to trials [17] are evidence of this reflexive and empirically driven direction in methodological research. As trials develop and change and focus on new areas of medicine, they will explore ever more complex interventions, using new methodological tools. As they do so, new and complex questions of conduct, policy and ethics will emerge.

New questions and methodological innovation allow important strides forward, as do shifts in our thinking about data and evidence. A move away from a deficit model, in which we see participants as “failing” to understand, allows us to use testimonies of participants and professionals on an equal footing, recognising and valuing the different expertise they have to offer. A widening of focus from initial recruitment to the full course of the trial allows us to consider the ongoing lived experience of participants into which trial-related events and views are incorporated. Interest in the “hidden challenges” [6] and the unseen areas of research practice allows close scrutiny of what Stobart [22] refers to as the “liminal spaces” in which the everyday work of trials is carried out. We need to explore these different components in order to improve our understanding of the whole.

This is a dynamic and evolving field, and we anticipate that the collective body of work for this Special Issue will clearly demonstrate the variety and complexity of the work we do. We hope that it will be a forum for methodological reflection and debate and will stimulate, in the spirit of qualitative enquiry, engagement with context, experience and expertise, and will bring to the fore new and emergent questions for clinical trials.
